# Epithelial barrier hypothesis in the context of nutrition, microbial dysbiosis, and immune dysregulation in metabolic dysfunction-associated steatotic liver

**DOI:** 10.3389/fimmu.2025.1575770

**Published:** 2025-05-14

**Authors:** Merve Cebi, Yusuf Yilmaz

**Affiliations:** ^1^ Department of Medical Biology, School of Medicine, Recep Tayyip Erdoğan University, Rize, Türkiye; ^2^ Department of Gastroenterology, School of Medicine, Recep Tayyip Erdoğan University, Rize, Türkiye; ^3^ The Global NASH Council, Washington, DC, United States

**Keywords:** metabolic dysfunction-associated steatotic liver disease, epithelial barrier hypothesis, gut-liver axis, nutrition, gut microbiota, inflammation

## Abstract

In recent years, the prevalence of chronic liver diseases, particularly Metabolic Dysfunction-Associated Steatotic Liver Disease (MASLD), has increased significantly. This upward trend is largely associated with lifestyle-related factors such as unhealthy dietary habits, physical inactivity, and various environmental influences. Among the key elements contributing to the pathogenesis of MASLD, the integrity of the intestinal epithelial barrier emerges as a critical determinant, given its central role in maintaining immune homeostasis along the gut-liver axis. Disruption of this barrier, often driven by excessive consumption of saturated fats and refined carbohydrates in combination with low dietary fiber intake, can lead to microbial dysbiosis. This imbalance in the gut microbiota triggers immune dysregulation and promotes systemic inflammation, thereby exacerbating hepatic injury. This review discusses the contribution of epithelial barrier dysfunction to the development and progression of MASLD, with a particular focus on how increased intestinal permeability may initiate and sustain chronic liver inflammation. Additionally, the influence of dietary and environmental factors on epithelial integrity, immune responses, and the inflammatory cascade is addressed. A better understanding of the complex interplay between gut barrier impairment, immune modulation, and liver pathology may offer valuable insights into MASLD pathophysiology and contribute to the development of more targeted therapeutic strategies.

## 1 Introduction

Metabolic Dysfunction-Associated Steatotic Liver Disease (MASLD) develops through complex interactions among metabolic, environmental, and immune factors, posing a growing global health challenge (1–3). MASLD includes various liver conditions, ranging from simple steatosis to metabolic dysfunction-associated steatohepatitis (MASH), cirrhosis, and hepatocellular carcinoma (4). While traditional research has primarily focused on lipotoxicity, oxidative stress, and inflammation, recent findings highlight the critical role of epithelial barrier integrity in the pathogenesis of MASLD (5, 6).

The pathogenesis of MASLD involves complex interactions between metabolic dysfunction, immune activation, and epithelial barrier integrity. A high fat diet is a well-recognized factor contributing to hepatic lipotoxicity, characterized by the accumulation of toxic lipid species in hepatocytes, which leads to cellular damage, oxidative stress, and apoptosis (7, 8). Moreover, dietary intake influences gut microbiota composition and intestinal permeability, with dysbiosis induced by a high fat diet facilitating the entry of microbial components, including endotoxins like lipopolysaccharides (LPS), into the systemic circulation (9). The disruption of microbial balance and increased LPS levels activate Toll-like receptors (TLRs) along with other innate immune pathways, driving inflammation and worsening liver damage (10, 11). Dysregulated immune responses continue to drive this cycle, with pro-inflammatory cytokines like TNF-α and IL-6 contributing significantly to the persistence of liver inflammation and the progression of fibrosis (12).

The epithelial barrier of the gut plays a central role in maintaining tissue homeostasis by preventing the passage of harmful substances and microorganisms from the intestinal lumen into the circulation. When this barrier is compromised, it can lead to changes in the gut microbiota, weakened intestinal integrity, and increased translocation of bacterial components such as endotoxins. These alterations contribute to liver inflammation (13, 14). Several environmental factors have been associated with epithelial barrier dysfunction. Among them, dietary composition, exposure to pollutants, and certain xenobiotics are known to disrupt barrier function, intensifying immune dysregulation and promoting persistent inflammation (15, 16).

In this review, the role of the epithelial barrier in MASLD is examined, with a focus on the mechanistic connections between intestinal permeability, immune activation, and liver pathology. By integrating current evidence, this review highlights how environmental and lifestyle factors compromise the integrity of epithelial barriers and the subsequent impact on the progression of metabolic liver disease.

## 2 Methods

This manuscript is a narrative review that aims to explore the role of epithelial barrier dysfunction in the pathogenesis of MASLD, with a focus on the interplay between nutrition, microbial dysbiosis, and immune dysregulation. A comprehensive literature search was conducted across four electronic databases: PubMed, Scopus, Google Scholar, and ScienceDirect. Studies published in English between 2000 and 2025, with a focus on publications from 2019 to 2025, were included. The search was performed using the following keywords and Boolean operators: (“Metabolic dysfunction-associated steatotic liver disease” OR “MASLD” OR “Non-alcoholic fatty liver disease” OR “NAFLD”) AND (“epithelial barrier dysfunction” OR “epithelial barrier integrity” OR “epithelial barrier hypothesis”) AND (“gut microbial dysbiosis” OR “gut microbiome dysregulation” OR “gut microbiota imbalance”) AND (“gut-liver axis”) AND (“nutrition”) AND (“immune dysregulation”) (**Table 1**). Articles were selected based on title and abstract screening, followed by full-text evaluation. Reference lists from these studies were manually searched for additional relevant publications. In this review, findings from human and animal studies are assessed separately to evaluate the potential mechanisms involved. This approach was used to evaluate the consistency of findings between the two models. Mechanisms observed in animal studies were also explored in human studies, which helped enhance the reliability and relevance of the results in both contexts. The synthesis of the data was conducted narratively, focusing on key themes related to epithelial barrier integrity, nutrition, microbial dysbiosis, and immune response in MASLD progression.

**Table 1:** Literature search strategy.

Criteria	Details
Databases Searched	PubMed, Scopus, Google Scholar, ScienceDirect
Publication Period	2000–2025, with a primary focus on publications from 2019–2025
Keywords	(“Metabolic dysfunction-associated steatotic liver disease” OR “MASLD” OR “Non-alcoholic fatty liver disease” OR “NAFLD”) AND (“epithelial barrier dysfunction” OR “epithelial barrier integrity” OR “epithelial barrier hypothesis”) AND (“gut microbial dysbiosis” OR “gut microbiome dysregulation” OR “gut microbiota imbalance”) AND (“gut-liver axis”) AND (“nutrition”) AND (“immune dysregulation”)
Language	English
Inclusion Criteria	Studies published in English, focusing on epithelial barrier dysfunction, nutrition, gut microbiota, immune dysregulation, and their roles in MASLD pathogenesis.
Exclusion Criteria	Studies not in English, unrelated to MASLD, or non-peer-reviewed articles (e.g., conference abstracts).
Data Synthesis	Human studies focused on clinical relevance to MASLD pathogenesis; animal studies explored mechanistic insights and therapeutic targets.

## 3 The epithelial barrier hypothesis: origins and development

The epithelial barrier hypothesis, first introduced in relation to allergic diseases, underscores the essential function of epithelial integrity in preserving immune balance and preventing pathological immune responses (17). This hypothesis suggests that exposure to various environmental stressors such as air pollutants, detergents, microplastics, nanoparticles, and dietary components can compromise the integrity of epithelial barriers, resulting in increased permeability, microbial dysbiosis, and immune dysregulation (18). While initially focused on respiratory and skin barriers, recent research highlights the significance of intestinal epithelial barrier dysfunction in the development of chronic inflammatory diseases, including MASLD (5, 19). Furthermore, studies suggesting an increased predisposition to allergic diseases in MASLD patients point to a potential parallel between immune dysregulation in metabolic and allergic diseases (20–22).

**Figure 1:**
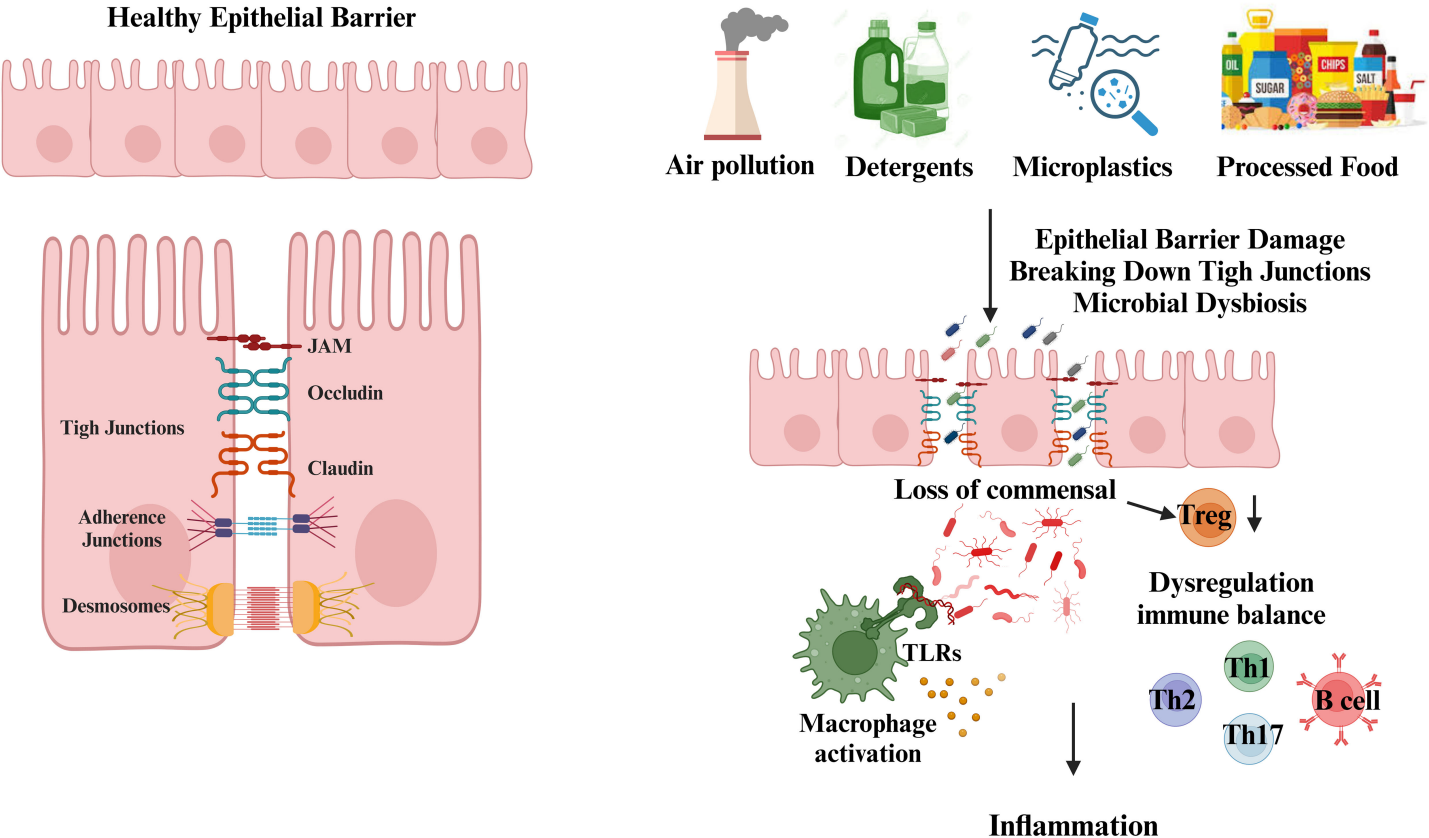
Disruption of the epithelial barrier by environmental toxic agents. Epithelial cells form protective barriers through the complex interaction of tight junctions (TJs), adherens junctions (AJs), and desmosomes. The tight junctions are formed by proteins such as claudins, occludins, and junctional adhesion molecules. Located beneath the tight junctions, adherens junctions contribute to cell-cell adhesion. Desmosomes, with their symmetrical structure, connect adjacent plasma membranes and are crucial for the stability and maintenance of cellular junctions. Exposure to environmental stressors, such as air pollutants, detergents, microplastics, nanoparticles, and dietary components, compromises epithelial barrier integrity. This leads to increased permeability, microbial dysbiosis, and immune dysregulation. Disruption of tight junctions (TJ), adherens junctions (AJ), and desmosomes allows the translocation of bacteria, endotoxins, and dietary antigens into systemic circulation. This breach activates innate immune responses via pattern recognition receptors, including Toll-like receptors (TLRs), triggering inflammatory cascades. Additionally, the loss of commensal Treg cell populations contributes to the dysregulation of Th1, Th2, Th17, and B cell immune responses, further promoting inflammation. (Created with BioRender.com).

Epithelial barriers function as a primary defense mechanism, creating both a physical and chemical shield against external insults. The structural stability of epithelial layers is maintained by cellular connections such as tight junctions (TJ), adherens junctions (AJ), and desmosomes, while mucosal secretions, antimicrobial peptides, and immunoglobulin A (IgA) provide additional protection (23). Compromise of this barrier function, commonly referred to as ‘leaky epithelium,’ allows the entry of gut-derived molecules into the bloodstream. These breaches in barrier integrity are implicated in triggering innate immune responses via pattern recognition receptors such as TLRs, which detect microbial components and initiate inflammatory cascades (**Figure 1**) (24, 25),.

The epithelial barrier hypothesis has evolved with growing recognition of the interplay between dietary patterns and epithelial integrity. Westernized diets, rich in saturated fats, refined sugars, and low in fiber, are linked to the disruption of intestinal epithelial integrity through alterations in gut microbiota composition and diversity (26, 27). Specifically, high-fat diets promote the overgrowth of pathobionts and reduce beneficial commensals, leading to increased bacterial endotoxin production and systemic inflammation (28). Additionally, lipotoxicity associated with metabolic dysfunction further compromises epithelial integrity by increasing oxidative stress and apoptosis in epithelial cells (29).

In the context of MASLD, the epithelial barrier hypothesis provides a compelling framework to understand the progression from metabolic dysfunction to liver inflammation. When intestinal barriers are compromised, microbial components like LPS can enter systemic circulation and activate hepatic Kupffer cells and stellate cells. This activation triggers the release of inflammatory cytokines and fibrogenic molecules, contributing to liver damage and fibrosis (30, 31). These immune-mediated processes contribute to the development of steatohepatitis, fibrosis, and ultimately cirrhosis in MASLD patients.

## 4 Gut-liver axis: bridging the intestinal and hepatic inflammation

The gut-liver axis, a crucial communication network connecting the gastrointestinal system and the liver, relies on anatomical connections and is involved in the regulation of immune responses, metabolic functions, and hormonal balance. The gut and liver are anatomically connected through the portal venous system. This system transports venous blood absorbed from the intestines to the liver, thereby delivering nutrients, microbial products, and potentially harmful substances. The liver processes these components by carrying out metabolic functions and facilitates the elimination of harmful compounds. Additionally, bile produced by the liver is delivered to the duodenum, where it contributes to digestion and plays a role in regulating the intestinal environment (32). This interaction is not limited to anatomical connections, but is also mediated through hormonal signals. Various hormones play a role in regulating the functions of both the gut and liver. For instance, cholecystokinin, secreted by endocrine cells in the gut, initiates bile production in the liver, while fibroblast growth factor-19 (FGF-19) regulates bile acid synthesis (33). Additionally, insulin-like growth factor (IGF), produced by the liver, helps maintain the integrity of the intestinal epithelium, contributing to the preservation of the intestinal barrier (34). The intestinal barrier is a critical factor that reinforces this complex relationship. While the gut facilitates the efficient absorption of nutrients and water, it is also responsible for preventing the passage of harmful microorganisms and toxins. The primary structural components of the barrier include epithelial cells, the mucus layer, microvilli, and associated proteins. These structures, through tight junctions between intestinal epithelial cells, limit paracellular permeability, thereby preventing the entry of harmful substances into the systemic circulation (35). The disruption of intestinal barrier function leads to the translocation of harmful microorganisms and their metabolic products to the liver. This condition impairs the liver’s ability to perform its normal functions and contributes to the initiation of pathological processes such as inflammation and fibrosis.

### 4.1 Gut microbiota and intestinal barrier in the gut-liver axis

The gut microbiome and the integrity of the intestinal epithelial barrier are essential for maintaining systemic balance. The intestinal barrier, consisting of tight junctions, antimicrobial peptides, and secretory immunoglobulin A, ensures tolerance to commensal microorganisms while preventing the entry of harmful pathogens and their products into the bloodstream (36). However, factors such as high-fat and low-fiber diets, alcohol consumption, overuse of antibiotics, chronic stress, and gastrointestinal infections can disrupt the gut microbiota, causing an alteration in the microbial balance (37). Dysbiosis is characterized by an overgrowth of gram-negative bacteria, which produce LPS. This bacterial endotoxin can compromise intestinal barrier integrity, resulting in increased permeability (“leaky gut”). The entry of LPS and additional microbial components into portal circulation triggers a cascade of inflammatory responses in the liver (38).

Leaky gut has emerged as a topic of growing scientific interest due to its reported involvement in a wide range of gastrointestinal and systemic conditions, including irritable bowel syndrome, various neurological conditions, asthma, and MASLD (39, 40). This increasing attention is also driven by the proposition that improving intestinal barrier function through dietary changes, probiotic supplementation, and other therapeutic approaches may offer potential benefits in the management of such diseases (41). In addition, it has been reported that the increase in intestinal permeability is associated with visceral adiposity and liver fat content in humans (42). Since visceral adiposity is also linked to other metabolic issues such as insulin resistance and high LDL levels, it suggests that the issue of “leaky gut” contributes to metabolic dysfunction.

The disruption of intestinal barrier integrity, commonly referred to as “leaky gut,” has been associated with a range of biomarkers that reflect bacterial translocation and immune activation. Among these, zonula occludens-1 (ZO-1), a structural component of tight junctions, has been reported to be significantly elevated in individuals with type 2 diabetes mellitus, reflecting increased intestinal permeability. In a study conducted on Asian Indian subjects, serum ZO-1 levels were markedly higher in patients with type 2 diabetes compared to healthy controls, and showed positive correlations with circulating LPS, inflammatory cytokines (TNF-α, IL-6), and markers of poor glycemic and lipid control. These findings support the use of ZO-1 as an early biomarker of impaired epithelial integrity and its potential role in the inflammatory and metabolic disturbances associated with insulin resistant states (43). Moreover, bacterial components such as LPS, peptidoglycan, and bacterial DNA have been detected in the bloodstream, pointing to microbial translocation from the intestinal lumen into systemic circulation (44, 45). These bacterial products are not only indicators of barrier dysfunction but also play a direct role in immune system activation. For instance, they stimulate the production of serum immunoglobulins IgG, IgA, and IgM which function as indirect biomarkers of chronic endotoxin exposure (46). In parallel, lipopolysaccharide-binding protein (LBP), an acute-phase protein primarily secreted by the liver, binds to LPS and facilitates its clearance. Its elevated levels in individuals with type 2 diabetes and morbid obesity suggest a state of ongoing low-grade inflammation and microbial translocation (47). However, recent human data indicate a more complex relationship between LBP and metabolic dysfunction. In a cohort of obese individuals, circulating LBP levels were found to be increased compared to non-obese controls, yet paradoxically, lower LBP levels were independently associated with type 2 diabetes and more advanced stages of hepatic steatosis and lobular inflammation. Furthermore, LBP concentrations were inversely correlated with markers of visceral adipose tissue inflammation. These findings imply that, while LBP is elevated in obesity, it may reflect a compensatory hepatic response to chronic caloric excess and serve a protective role by enhancing hepatic LPS clearance, thereby attenuating the metabolic consequences of obesity (48). Similarly, bactericidal/permeability-increasing protein (BPI), produced by neutrophils, neutralizes LPS and thereby mitigates its pro-inflammatory effects, indicating its role as both a defensive molecule and a biomarker of innate immune activity (49). In patients with liver cirrhosis, elevated plasma BPI levels and increased neutrophil BPI mRNA expression have been observed, particularly in those with advanced disease stages. Moreover, BPI levels were positively correlated with TNF-α concentrations, and *in vitro* experiments demonstrated that higher circulating BPI significantly attenuated LPS-induced TNF-α production by monocytes. These findings suggest that BPI not only reflects enhanced innate immune activation in the context of gut-derived endotoxemia but also functions as a negative regulator of LPS-mediated inflammatory signaling (50). Furthermore, soluble CD14, which reflects monocyte activation, binds to the LPS-LBP complex and mediates its transfer to the TLR4/MD2 signaling pathway. This interaction triggers NF-κB activation and the release of pro-inflammatory cytokines, thereby amplifying systemic immune responses (51). Importantly, elevated levels of circulating sCD14 have been shown to correlate with histological severity of liver inflammation in patients with MASLD. In a liver biopsy-confirmed cohort, serum sCD14 levels were significantly associated with MASH diagnosis, MASLD activity score, and hepatic CD14 mRNA expression, independently predicting the degree of hepatic inflammation (52). Supporting these observations, another study using both murine models of diet-induced MASH and clinical samples from biopsy-proven MASLD patients demonstrated that sCD14 levels were significantly higher in individuals with MASH compared to those with simple steatosis. Moreover, sCD14 was positively correlated with histological features such as lobular inflammation, hepatocellular ballooning, and fibrosis (53).

The extent to which biomarkers associated with impaired intestinal barrier function correlate with metabolic health parameters remains an important yet insufficiently addressed research question. To help fill this knowledge gap, a recent study evaluated gut permeability-related biomarkers that can be measured in serum and plasma samples and assessed their relationship with metabolic health status. The study selected a total of 80 individuals from a previously conducted large-scale cohort (n = 2048), based on extreme values of waist circumference, fasting glucose, LDL cholesterol, and gamma-glutamyl transferase (GGT) levels, representing the most metabolically healthy and unhealthy profiles. Eight leaky gut-related markers identified from the literature were analyzed in serum or EDTA-plasma samples obtained from these individuals. The findings demonstrated that levels of zonulin, lipopolysaccharide-binding protein (LBP), soluble CD14 (sCD14), bactericidal/permeability-increasing protein (BPI), and peptidoglycan were significantly elevated in participants with poor metabolic profiles. In contrast, no significant differences were observed between the groups in terms of EndoCAb IgA, IgG, and IgM levels. Stepwise regression analyses revealed that zonulin, in particular, showed strong associations with body mass index, fasting glucose, triglycerides, GGT, and C-reactive protein levels. These findings suggest that zonulin may serve as a primary biomarker for monitoring intestinal barrier dysfunction and identifying individuals at increased risk for metabolic diseases (54).

### 4.2 LPS-mediated immune activation in the gut-liver axis

LPS, an important molecule found in the outer membrane of gram-negative bacteria, significantly contributes to the disruption of the gut-liver axis communication. When intestinal permeability is compromised, LPS reaches the liver through the portal circulation, triggering activation of Kupffer cells and various other immune cells within the liver via TLRs (9). This activation triggers downstream signaling pathways, such as MyD88 and NF-κB, resulting in the release of pro-inflammatory cytokines (55). These cytokines amplify hepatic inflammation and recruit additional immune cells, including neutrophils and monocytes, to the liver.

The role of LPS mediated immune responses in the pathogenesis of MASLD is supported by both clinical observations and experimental data. Clinically, elevated serum LPS levels have been strongly associated with the progression of liver disease. Notably, a population-based study reported that individuals with the highest LPS levels accounted for approximately 30% of liver-related complications (56).

Experimental evidence further substantiates these findings. Chronic exposure to LPS induces sustained hepatic inflammation, hepatocyte apoptosis, and hepatic stellate cell activation, all of which collectively promote fibrosis and cirrhosis (57–62). Moreover, animal models with gut microbiota alterations exhibit increased susceptibility to LPS-induced liver injury, highlighting the contribution of intestinal dysbiosis to immune dysregulation. Several studies have demonstrated that interventions targeting the gut microbiota—such as polyphenols from Aronia melanocarpa (63), rhein (64), Lactobacillus paracasei CCFM1223 (65), and sulforaphane (66) can protect against LPS-induced hepatic injury by modulating the gut-liver axis. In addition, host microbiota composition has been shown to determine the extent of LPS-driven hepatic inflammation (67).

One of the other key mechanisms of LPS mediated hepatic inflammation is the indirect regulation of TREM1 and TREM2 expression through the TLR4 signaling pathway activated by LPS. During LPS exposure, which leads to a systemic inflammatory response, it has been reported that TREM1 expression is increased in hepatic macrophages and endothelial cells, while TREM2 expression is decreased (68). TREM1 enhances the expression of inflammatory genes such as MyD88, CD14, and IL-1β, triggering the release of TNF-α and IL-1β and activating transcription factors like AP-1 and NF-κB (69, 70). On the other hand, TREM2 expression is inhibited by TNF-α, and its levels are significantly reduced under acute inflammatory conditions (68, 71). However, this dynamic may differ in chronic liver injury. In chronic inflammation, Kupffer cells have been reported to transform into anti-inflammatory M2 macrophages, thereby protecting the liver from chronic inflammation (72). In summary, TREM1 and TREM2 play different roles in inflammation processes. While TREM1 exerts pro-inflammatory effects during acute inflammation, TREM2 generates a regulatory response under conditions of excessive inflammation, thereby protecting hepatic tissue from damage.

Recently, the close association of TREM2 with insulin resistance and type 2 diabetes, as well as its mechanistic role in MASLD, has attracted considerable attention (73, 74). Also, it is known that 20% of MASLD patients progress to the MASH stage with chronic inflammation and fibrosis, which can lead to cirrhosis and HCC, with TREM2+ macrophages playing a crucial role in this process. In a chemical cholestasis mouse model, TREM2 has been reported to exert a protective effect by reducing liver inflammation and preventing hepatocyte injury (75). Studies in animal models have demonstrated that Trem2^−^/^−^ mice develop insulin resistance, glucose intolerance, adipocyte hypertrophy, and lipid accumulation under diet-induced obesity conditions. Notably, these mice fail to form crown-like structures (CLS), which play a critical role in the clearance of apoptotic adipocytes and lipid debris during obesity (74, 76, 77). Moreover, it has been reported that Trem2^−^/^−^ mice fed a long-term high fat diet develop more severe hepatic steatosis (77). TREM2 is an important receptor that regulates the transition of macrophages from an inflammatory phenotype to a repair and regeneration focused phenotype following liver injury. In a study using acute (acetaminophen, APAP) and chronic (carbon tetrachloride, CCl_4_) hepatotoxic injury models, it has been shown that in Trem2^−^/^−^ mice, compared to wild-type mice, higher levels of pro-inflammatory cytokines (IL-6, TNF-α) and chemokines (MCP-1) are released from Kupffer cells and hepatic stellate cells. This increase is reported to lead to more severe liver damage. In contrast, in wild-type mice where TREM2 expression is maintained, inflammation remains more limited, and the recovery process begins more rapidly after injury. During the recovery phase, it has been reported that macrophages transitioning into Kupffer cells express high levels of TREM2 and exhibit a unique transcriptomic profile that is sensitive to oxidative stress and suppresses pro-inflammatory signals (72, 78).

In addition to animal studies, human data also provide valuable insights into the role of TREM2 in the progression of MASLD. The liver tissue obtained from the MASH patient group and the control group with hepatic hemangioma, which is known not to have been diagnosed with MASH, shows that the upregulation of TREM2 in MASH patients is associated with hepatic steatosis and inflammation. However, this upregulation is suggested as a protective effect. Additionally, it is highlighted in this study, based on an animal model, that TREM2 promotes the resolution of inflammation by providing immune modulation via TGFβ1 (79). On the other hand, recently, the soluble form of TREM2 (sTREM2) has attracted attention due to its elevated levels in MASH patients (80). It has been suggested that plasma sTREM2 levels in MASH patients correlate with ALT and AST, are associated with MASH risk status, and may serve as a reasonable biomarker for identifying patients eligible for clinical trials in MASH (80, 81). However, data regarding the mechanism of action of sTREM2 in the pathogenesis of MASLD is limited. While it has been reported that sTREM2 may have differential effects on macrophage cytokine expression *in vitro* (82), *in vivo* studies validating these findings are still lacking. Furthermore, in a recent study, preoperative and postoperative plasma sTREM2 levels were measured in 108 patients undergoing liver resection. The results showed that preoperative sTREM2 levels were associated with advanced-stage liver fibrosis and post-hepatectomy liver failure. Compared to traditional parameters such as FIB-4, MELD, Child-Pugh, and LiMAx, sTREM2 demonstrated higher accuracy in predicting post-hepatectomy liver failure. These findings suggest that TREM2 may be a clinically valuable biomarker for predicting early-stage liver diseases and postoperative complications (83).

A recent study has reported that the function of TREM2 is directly related to nutritional status. Chronic liver inflammation induced by obesity is a hallmark of MASH, an aggressive form of MASLD. However, the exact mechanism by which this low grade, yet persistent inflammation is sustained in the liver remains unclear. The animal study demonstrates that TREM2, a macrophage phagocytic receptor induced by sphingosine-1-phosphate derived from hepatocytes, plays a crucial role in the clearance of lipid-laden apoptotic cells, thereby maintaining liver immune homeostasis. However, prolonged overnutrition increases the production of pro-inflammatory cytokines (such as TNF and IL-1β) in the liver, leading to the loss of TREM2 function. This process occurs through the proteolytic cleavage of TREM2 by an enzyme called ADAM17. The loss of TREM2 impairs the proper clearance of dead hepatocytes, resulting in their abnormal accumulation. Consequently, this accumulation may trigger further inflammation, accelerating the transition to MASH in a vicious cycle (84).

### 4.3 Microbial metabolites and immune regulation in the gut-liver axis

Microbial metabolites, particularly short-chain fatty acids (SCFAs) including acetate, propionate, and butyrate, are essential in influencing the gut-liver axis by regulating metabolic and immune responses (85). These metabolites, generated through the digestion of dietary fibers by gut microbes, show significant anti-inflammatory effects (86). SCFAs modulate immune activity by reducing the secretion of inflammatory molecules and promoting the strength of the intestinal barrier (87–90).

The beneficial effects of SCFAs are mediated through multiple mechanisms. A primary mechanism involves the stimulation of G protein-coupled receptors (GPCRs), including GPR41 and GPR43, which are found on various immune cells. Activation of these receptors leads to the suppression of immune cell activation and a balanced regulation between inflammatory and tolerogenic responses (91, 92). Additionally, butyrate plays a key role in modulating gene expression by inhibiting histone deacetylases (HDACs), enzymes responsible for the removal of acetyl groups from histones, thereby influencing chromatin structure and immune cell function (93). By inhibiting HDACs, butyrate promotes chromatin decondensation, increasing its accessibility and enabling changes in gene expression. This epigenetic modulation alters the transcriptional activity of immune cells, promoting anti-inflammatory pathways and diminishing the release of inflammatory factors (91, 94, 95). As a result, the immune response is reprogrammed to favor tolerance over inflammation, contributing to the suppression of excessive immune activation.

SCFAs significantly influence immune cell function, particularly in T and B cells. For example, butyrate encourages the development of regulatory T cells (Tregs), crucial for sustaining immune balance and controlling inflammation, primarily by increasing histone acetylation at the FoxP3 locus (96, 97). SCFAs also influence the function of effector T cells, including Th1 and Th17 subsets, through HDAC inhibition, leading to increased mTOR activity and enhanced cytokine production (98, 99). In antiviral immunity, SCFAs, especially butyrate, improve CD8+ T cell memory formation, facilitating a more robust immune response (100, 101). In addition, SCFAs promote the conversion of B cells into plasma cells, enhancing antibody production through metabolic pathways that support glycolysis and fatty acid synthesis (102, 103). These effects are mediated by HDAC inhibition, which alters gene expression related to B cell differentiation (104, 105). Furthermore, SCFAs impact T cell activity, especially T follicular helper (Tfh) cells, which play a secondary role in modulating B cell function and antibody production (106). SCFAs also support the differentiation of regulatory B cells (B10 cells), contributing to immune homeostasis by promoting acetylation processes (107, 108).

However, when the gut microbiota is disrupted, as seen in dysbiosis, SCFA production is significantly reduced. This reduction in SCFAs disrupts the gut-liver axis and immune regulation, resulting in heightened intestinal permeability. As a result, harmful bacteria and their byproducts are able to enter the bloodstream, worsening systemic inflammation and liver damage (109). The reduction in SCFA production disrupts the immune-regulatory capacity of both the gut and liver, thereby exacerbating inflammatory pathways and impairing anti-inflammatory responses. This imbalance is involved in the initiation and progression of liver disorders like MASLD, primarily through enhancing liver inflammation and sustaining liver damage (110–112). The understanding of this intricate interplay between microbial metabolites, immune regulation, and liver disease progression opens new avenues for therapeutic interventions targeting microbial metabolites or their pathways to restore immune homeostasis and mitigate liver pathology.

## 5 Impact of diet on gut barrier integrity in MASLD

**Figure 2:**
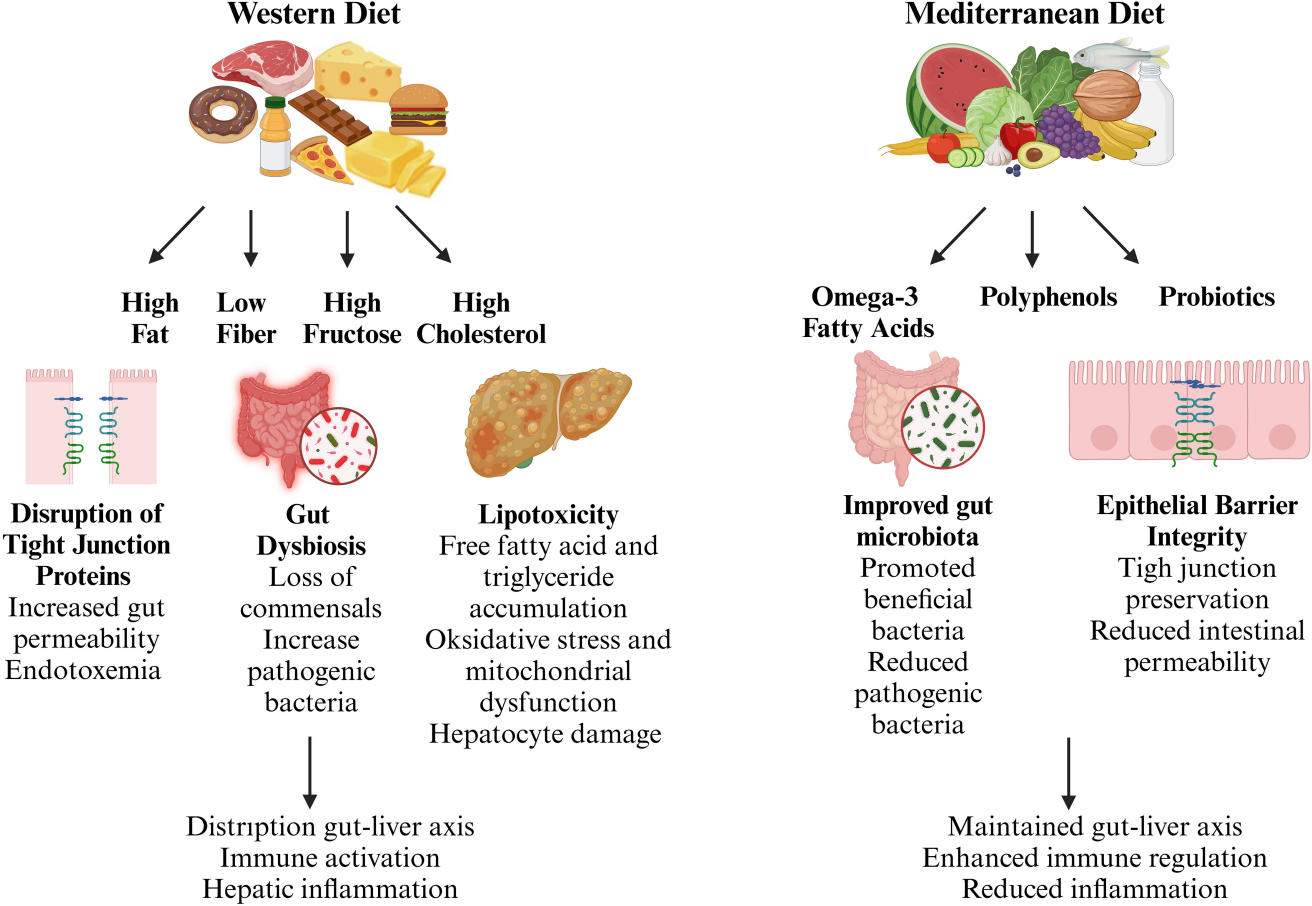
Dietary modulation of gut-liver axis in metabolic dysfunction-associated steatotic liver disease (MASLD). The gut-liver axis plays a pivotal role in the pathogenesis of MASLD, with dietary components acting as key modulators of gut homeostasis and immune regulation. A Western diet, characterized by high fat, low fiber, and excessive fructose intake, disrupts intestinal barrier integrity, alters gut microbiota composition, and promotes systemic inflammation, contributing to hepatic injury. In contrast, bioactive dietary components such as omega-3 fatty acids, polyphenols, and probiotics enhance gut barrier function, support a beneficial microbiota, and mitigate inflammatory responses. (Created with BioRender.com).

Dietary factors, intestinal health, and liver function are closely linked and contribute significantly to the development of MASLD. The gastrointestinal tract acts as the primary barrier to harmful dietary and microbial substances, and its proper function is crucial for preserving metabolic stability. However, modern dietary habits characterized by high-fat, low-fiber, and fructose-rich foods can disrupt gut homeostasis, alter microbiota composition, and compromise the gut barrier, ultimately fueling systemic inflammation and liver injury. On the other hand, bioactive substances like omega-3 fatty acids, polyphenols, and probiotics demonstrate protective properties by influencing gut-liver communication (**Figure 2**). This section explores the complex relationship between dietary components and gut barrier function, shedding light on their implications for MASLD pathogenesis and potential dietary interventions.

### 5.1 High-fat diets

High-fat diets, especially saturated fats (e.g., red meat, butter, full-fat dairy) and industrially produced trans fats (e.g., processed and fast foods), are strongly linked to the progression of metabolic dysfunction, including MASLD. These diets disrupt the gut-liver axis through various interconnected mechanisms, contributing to increased intestinal permeability, microbiota dysbiosis, and systemic inflammation, all of which exacerbate liver damage.

Experimental studies conducted using animal models have demonstrated that high-fat diets impair epithelial barrier integrity at an early stage. Specifically, one study showed that a high-fat diet induces disruption of the paracellular barrier in the proximal small intestine before the onset of type 2 diabetes or endotoxemia. This disruption was associated with a reduction in the levels of tight junction proteins such as claudin-1, occludin, and ZO-1 (113). This barrier disruption is supported not only by molecular changes but also by structural alterations. A study conducted on middle-aged mice revealed that after 14 weeks of a high-fat diet, there was a reduction in the length of small intestine villi and the depth of colon crypts, along with impaired colon barrier function. These findings indicate that the epithelial structure is compromised both morphologically and functionally. The study highlights that such a diet may have lasting effects on epithelial integrity (114). Additionally, a study investigating the acute effects of high-fat diets on intestinal injury revealed that short-term exposure to high-fat feeding exacerbated intestinal damage by impairing the clearance of dead neutrophils by macrophages. This dysfunction in macrophage activity hindered the activation of repair pathways, including the production of IL-10, which is crucial for resolving inflammation and promoting barrier repair. These findings suggest that high-fat diets not only disrupt epithelial barrier integrity but also interfere with immune cell-mediated repair mechanisms, preventing proper recovery of the intestinal barrier (115).

The effects of high-fat diets on intestinal epithelial barrier integrity not only trigger local inflammation but also facilitate the passage of microbial products, particularly endotoxins such as LPS, into the systemic circulation, potentially leading to endotoxemia. High-fat diets increase intestinal permeability, allowing LPS to be transmitted to the immune system. Experimental studies have demonstrated that mice fed high-fat diets exhibit heightened sensitivity to LPS, with inflammatory responses being aggravated through the activation of TLR-4, a key receptor involved in mediating immune responses to endotoxins (116). In parallel, human studies have elucidated the role of LPS influx in metabolic dysfunctions. A study conducted on Japanese males revealed a significant relationship between the daily influx of LPS, originating from gut bacteria, and various metabolic parameters. Notably, fasting plasma LPS concentrations were inversely correlated with perceived fullness, whereas a positive correlation was found with carbohydrate intake. Additionally, LPS levels were associated with fasting breath acetone, a biomarker of fat metabolism, suggesting that LPS influx may influence metabolic processes even in the absence of significant changes in body weight (117). The effects of high-fat diets on LPS-associated inflammation have also been reported in metabolic diseases such as polycystic ovary syndrome (PCOS). A study conducted on women with PCOS has reported that a high-fat diet increases circulating levels of LPS and TLR-4 (118).

High-fat diets also contribute to lipotoxicity in hepatocytes through excessive accumulation of free fatty acids (FFAs), leading to cellular damage (119). Experimental studies using hepatocyte cultures treated with palmitic acid, a predominant saturated fat, have shown elevated levels of reactive oxygen species (ROS) and the initiation of apoptosis pathways through mitochondrial dysfunction and endoplasmic reticulum (ER) stress (120–122). Additional evidence highlights that palmitic acid exposure significantly elevates ER stress markers such as CHOP and GRP78, further contributing to hepatocyte dysfunction and injury (123). *In vivo* studies in animal models, where mice were fed a high-fat diet containing 60% fat for 12 weeks, show significant hepatic triglyceride accumulation, alongside the activation of pro-inflammatory signaling pathways like NF-κB and JNK (124). These pathways trigger the production of cytokines like TNF-α and IL-6, which exacerbate hepatocyte apoptosis and worsen liver inflammation. Interestingly, genetic knockout models for NF-κB signaling have demonstrated reduced inflammation and hepatocyte injury, underscoring the critical role of this pathway in disease progression (125). In human studies, the mechanisms observed in animal models are also evident in patients with Metabolic Dysfunction-Associated Steatotic Liver Disease (MASLD). Liver biopsies from MASLD patients reveal elevated ROS levels, ER stress markers, and increased NF-κB activation (126–128).

### 5.2 Low-fiber diets

Dietary fiber, primarily sourced from whole grains, fruits, vegetables, and legumes, supports microbial diversity in the gut and helps maintain gut barrier integrity. A low-fiber diet, often characteristic of Western dietary patterns, can have detrimental effects on both gut health and overall metabolic function.

Clinical and observational studies have consistently demonstrated that low-fiber diets contribute to gut dysbiosis, characterized by a decline in beneficial commensals such as *Bifidobacterium* and *Lactobacillus*, alongside an increased abundance of pro-inflammatory taxa, including *Escherichia* and *Streptococcus* (129, 130). This microbial imbalance can exacerbate gut inflammation, which is a key contributor to the systemic inflammation observed in MASLD. The disruption of gut homeostasis and immune modulation can worsen liver injury, accelerating the progression of fatty liver and fibrosis.

In MASLD patients, compositional shifts in the gut microbiota include an overrepresentation of pro-inflammatory genera such as *Dorea* and *Bilophila*, and a reduced abundance of anti-inflammatory taxa like *Faecalibacterium*, *Akkermansia*, and *Alistipe* (131–133). These microbial alterations are associated with changes in liver function. For example, *Dorea* abundance correlates with elevated liver enzymes, including aspartate aminotransferase (AST) and alanine aminotransferase (ALT), suggesting a potential role in liver injury, whereas *Alistipes* appears inversely related to serum glucose and ALT, indicating a possible protective effect (131). Moreover, alterations in gut microbiota are linked to the progression of liver fibrosis. Microbiota profiles in patients with advanced liver fibrosis show increased proportions of *Bacteroides*, while *Lactobacillus* and *Bifidobacterium* are less prevalent (134). Specific species, including *Bacteroides* and *Streptococcus*, are associated with fibrotic changes, while *Escherichia* and *Shigella* are strongly correlated with advanced fibrosis (135, 136).

Another significant consequence of low-fiber diets is the reduction in SCFAs synthesis, which are essential metabolites produced by gut microbes during the fermentation of fiber. Animal studies provide mechanistic insights into how low-fiber diets may contribute to disease development. In murine models, fiber deficiency leads to decreased production of short-chain fatty acids (SCFAs), which are crucial microbial metabolites generated during fiber fermentation (137, 138). Experimental studies have demonstrated that mice on low-fiber diets exhibit severe inflammatory responses in models of colitis, allergic airway disease, and food allergy, partly due to the loss of SCFA-mediated immune regulation (137, 139–141). SCFAs play an important role in maintaining gut barrier integrity, regulating immune responses, and supporting metabolic homeostasis. In mice, reduced SCFA levels are associated with increased intestinal permeability, higher endotoxin translocation, and chronic inflammation factors that may contribute to liver damage (142).

These findings emphasize the importance of dietary fiber in protecting gut and liver health. Low-fiber Western diets reduce SCFA production, increase gut permeability, and promote chronic inflammation factors that contribute to the development and progression of MASLD. In contrast, fiber-rich diets help maintain microbial balance, support immune regulation, and are associated with lower rates of liver-related complications (143, 144). Increasing fiber intake may be a simple but effective approach to reduce the risk of MASLD.

### 5.3 High fructose diets

Fructose, a naturally occurring monosaccharide found in fruits, has become a prominent component of modern diets through its industrial use as high-fructose corn syrup (HFCS), especially in sugary beverages and processed foods. While moderate fructose intake through whole fruits appears to have minimal metabolic impact (145), excessive consumption of processed fructose in mice has been implicated in the pathogenesis and progression of MASLD (146). Unlike glucose, fructose metabolism occurs largely in the liver and bypasses the regulatory steps controlled by insulin, thereby favoring unregulated *de novo* lipogenesis and hepatic triglyceride accumulation (147). These mechanisms contribute to insulin resistance, lipotoxicity, and hepatocellular stress, all of which promote MASLD development (148). Clinical studies have also shown that individuals with MASLD tend to consume more fructose than healthy controls, further reinforcing the association between high dietary fructose intake and disease progression (149, 150). Furthermore, epidemiological data suggest that regular consumption of fructose-rich soft drinks is associated not only with hepatic steatosis and fibrosis but also with increased cardiovascular risk, even in the absence of metabolic syndrome (149–151). Beyond its direct effects on liver metabolism, fructose consumption also influences gut microbiota composition, which plays a crucial role in metabolic health. Animal studies have shown that high-fructose diets alter the *Firmicutes/Bacteroidetes* ratio, favoring the proliferation of bacteria associated with inflammation and liver fat accumulation (152, 153).

The disruption of the intestinal epithelial barrier by high-fructose diets is a key mechanism in the development of metabolic dysfunction. Several animal studies have demonstrated that excessive fructose intake impairs the intestinal barrier, leading to an increase in gut permeability. In mice, a high-fructose diet exacerbated colonic inflammation and intestinal damage, particularly in the context of chemically induced colitis. Fructose consumption reduced the expression of tight junction proteins such as occludin, while increasing pro-inflammatory cytokines, including IL-6, IL-1β, and TNF-α (154). Similarly, in rats, a dose-dependent study revealed that high fructose intake reduced the expression of key tight junction proteins (e.g., ZO-1, occludin), mucus layer components (e.g., MUC2, TFF3), and induced colonic inflammation. This was accompanied by an upregulation of IL-6 and IL-8 and a suppression of the anti-inflammatory cytokine IL-10. Additionally, microbial changes were observed, including a reduction in the abundance of beneficial bacteria such as *Lactobacillus* and *Blautia*, along with decreased production of SCFAs, indicating impaired intestinal barrier function (155). In piglets, which serve as a more physiologically relevant model for humans, 35 days of fructose supplementation resulted in the downregulation of tight junction-related genes in the ileum and colon. While overt inflammation was not observed, significant alterations in gut microbiota composition were noted, including an increase in *Streptococcus* and *Faecalibacterium* in the ileum and *Blautia* and *Clostridium sensu stricto 1* in the colon. These findings suggest that the dysfunction of the intestinal barrier may precede overt inflammation (156). Furthermore, a study that combined both animal and human data demonstrated the critical role of JAM-A in maintaining gut barrier integrity. In mice with genetic disruption of JAM-A, a high-fat, fructose, and cholesterol diet led to more severe steatohepatitis, characterized by increased gut permeability to endotoxins, disruption of tight junctions, and intestinal inflammation. In the same study, reduced JAM-A expression was also observed in colon biopsies from patients with MASLD, where it was associated with mucosal inflammation and absent in healthy individuals, suggesting that the gut-liver axis mechanisms identified in mice may also be relevant in humans (157).

Recent research has highlighted the role of gut microbiota in modulating immune responses, particularly T cell balance, in the development of hepatic steatosis following fructose consumption. A study in mice revealed that high-fructose diets induced an imbalance in T cell populations, with a significant increase in pro-inflammatory Th1 cells and a decrease in regulatory T (Treg) cells. This shift in immune balance was accompanied by increased colonic inflammation and intestinal damage (158). Moreover, the gut microbiota plays a pivotal role in the modulation of systemic inflammation and liver metabolism in fructose-induced metabolic disturbances. Fecal microbiota transplantation from high-fructose diet-fed mice into recipient animals was shown to alleviate systemic metabolic dysfunction, suggesting that restoring gut microbiota balance may help maintain liver and intestinal immune homeostasis (159).

### 5.4 High cholesterol diets

Cholesterol, an essential lipid predominantly obtained from animal derived foods such as yolks, shellfish, red meats, pork, and various dairy products, has drawn considerable attention for its role in the progression of MASLD. While cholesterol is necessary for numerous cellular functions, excessive intake especially from foods rich in cholesterol has been associated with the advancement of MASLD. Studies indicate that individuals with elevated cholesterol intake have a higher risk of developing fatty liver and experiencing more severe liver complications, highlighting the need for dietary changes to mitigate further liver damage (160).

Excessive cholesterol intake directly contributes to hepatic lipid accumulation, a hallmark of MASLD. Studies have demonstrated that high cholesterol consumption induces steatotic changes in hepatocytes, leading to metabolic dysregulation and cellular stress. Cholesterol-induced lipotoxicity disrupts mitochondrial function, impairing oxidative phosphorylation and energy balance, which contributes to the progression of liver damage and inflammation. Experimental studies in murine models have shown that high cholesterol diets increase liver weight and promote inflammatory responses in liver tissues (161, 162). Furthermore, experimental studies in murine models have shown that high cholesterol diets result in increased liver weight and elevated serum levels of leptin and IL-6, which are indicative of the inflammatory response in liver tissues (161). Furthermore, a separate murine model of MASH fed a high fat, cholesterol, and cholic acid diet demonstrated that after 1 week, mice exhibited increased oxidative stress, liver steatosis, and systemic inflammation, which worsened after 3 weeks of diet induction (162). Moreover, cholesterol accumulation within hepatocytes promotes lipotoxicity, contributing to hepatocyte dysfunction and apoptosis. This process is largely mediated by oxysterols, toxic cholesterol derivatives that impair mitochondrial function and disrupt cellular energy production. Mitochondrial dysfunction, in turn, exacerbates liver injury and facilitates progression from simple steatosis to more advanced stages of MASLD. Additionally, the presence of cholesterol crystals in the liver has been shown to damage hepatocytes and activate inflammatory signaling pathways, including hypoxia-inducible factor 1-alpha (HIF-1α), thereby aggravating liver inflammation and fibrosis (163, 164).

Dietary cholesterol contributes not only to disruptions in hepatic lipid metabolism but also to the development of insulin resistance, a critical factor in MASLD progression. Evidence from animal studies indicates that excessive cholesterol intake impairs hepatic insulin signaling, which leads to fat accumulation in liver tissue and promotes metabolic dysfunction. Under insulin-resistant conditions, the liver continues to drive lipid synthesis while inadequately suppressing glucose production, contributing to metabolic imbalances such as hyperglycemia and hypertriglyceridemia features commonly observed in MASLD (165). Additionally, experimental models have demonstrated that high-fat, high-cholesterol diets rapidly induce liver inflammation, steatosis, and fibrosis, with significant impacts on liver function and immune cell populations. The use of a high-fat, high-cholesterol diet in murine models has been shown to lead to liver inflammation and MASH within a short timeframe, providing a rapid method to evaluate potential therapeutic agents for MASLD (166).

In addition to its direct impact on hepatic lipid metabolism, dietary cholesterol also influences gut microbiota composition, which contributes to systemic inflammation and hepatic injury. High cholesterol diets in animal models have been shown to alter the gut microbiota, increasing the abundance of pro-inflammatory bacteria such as Mucispirillum and Desulfovibrio, while reducing beneficial microbes such as Bifidobacterium and Bacteroides. This microbial imbalance has been linked to increased intestinal permeability, allowing endotoxins to enter the bloodstream and induce systemic inflammation. The resultant inflammation exacerbates liver injury and accelerates the progression of MASLD (167, 168). Moreover, experimental studies have shown that a high-cholesterol diet aggravates intestinal barrier dysfunction, as evidenced by studies in murine models that demonstrate significant disruption in tight junction proteins, such as occludin and ZO-1. These disruptions are mediated by the modulation of sterol regulatory element-binding protein 2 (SREBP2), which regulates the endocytic degradation of tight junction proteins, leading to greater gut permeability (169). Interestingly, the activation of the inflammasome by cholesterol intake further contributes to intestinal inflammation. Studies on both mice and zebrafish have shown that dietary cholesterol triggers acute inflammatory responses in the intestine, marked by the activation of IL-1β and recruitment of myeloid cells. This local inflammation initiates a cascade of immune responses that exacerbate gut permeability and contribute to the translocation of endotoxins into systemic circulation, which subsequently worsens liver injury (170).

### 5.5 Omega-3 fatty acids

Omega-3 fatty acids, found in fatty fish like salmon and mackerel, as well as walnuts and flaxseeds, support hepatic lipid metabolism, reduce inflammation, and regulate fat metabolism. Meta-analyses suggest that fish oil-derived omega-3 fatty acids show promise in MASLD management, indicating their potential as a therapeutic strategy (171). Omega-3 fatty acids protect against hepatic steatosis by regulating lipogenesis and inflammation. In a murine model of total parenteral nutrition-induced liver injury, omega-3 supplementation reduced fat accumulation and improved liver function, with enteral administration showing the most significant protective effect (172).

Several meta-analyses further support the therapeutic effects of omega-3 polyunsaturated fatty acids (PUFAs) in MASLD. One study demonstrated that omega-3 PUFA supplementation significantly reduced hepatic lipid accumulation and AST levels, although its impact on ALT was not statistically significant (173). Another meta-analysis involving 10 randomized controlled trials with 577 MASLD patients found that omega-3 PUFAs reduced liver fat content, gamma-glutamyl transferase (GGT) levels, triglycerides (TG) and increased high-density lipoprotein (HDL). However, they did not show a significant impact on ALT, AST, total cholesterol (TC), or low-density lipoprotein (LDL) levels (174). A recent clinical study in individuals with type 2 diabetes and MASLD showed that 12 weeks of omega-3 supplementation resulted in notable decreases in liver fat accumulation, lipid storage markers, and abdominal fat levels (175). Additionally, a large UK Biobank cohort study found that omega-3 supplementation was linked to a reduced risk of liver diseases, such as MASLD, alcoholic liver disease, and liver failure, with more pronounced effects in women and individuals carrying the PNPLA3 rs738409 risk allele (176). These findings suggest that omega-3 PUFAs may help reduce hepatic lipid accumulation and metabolic dysfunction in MASLD, highlighting the need for further clinical trials to establish optimal dosing and long-term efficacy.

Omega-3 polyunsaturated fatty acids (PUFAs) have been implicated not only in hepatic metabolic regulation but also in the modulation of the gut-liver axis, particularly through their impact on intestinal barrier function. Evidence from human studies indicates an inverse association between omega-3 intake and serum zonulin concentrations, suggesting a potential role in enhancing epithelial barrier integrity (177). In a sub-analysis of the LIBRE clinical trial, greater adherence to a Mediterranean diet enriched in omega-3 fatty acids was linked to elevated plasma levels of docosahexaenoic acid (DHA), alongside reductions in plasma lipopolysaccharide-binding protein (LBP) and fecal zonulin both markers associated with intestinal permeability. While these improvements were statistically significant, their magnitude was somewhat less pronounced compared to the effects attributed to short-chain fatty acid (SCFA) production (178).

Complementary findings from preclinical studies have further elucidated the mechanisms by which omega-3 PUFAs exert their barrier-protective effects. In senescence-accelerated mouse prone 8 (SAMP8) models, chronic dietary supplementation with eicosapentaenoic acid (EPA) and DHA mitigated age-associated increases in intestinal permeability, partially through restoration of microbial diversity and enrichment of SCFA-producing bacteria, thereby enhancing epithelial integrity (179). Similar effects were observed in mice fed a high-fat diet, where omega-3 intake preserved tight junction architecture, increased goblet cell numbers, and attenuated colonic and systemic inflammation by suppressing TLR4/NF-κB signaling pathways (180). Moreover, in a rat model of obstructive jaundice, omega-3 treatment improved mucosal epithelial morphology and goblet cell density while reducing inflammation via downregulation of the HMGB1/TLR4/NF-κB axis, ultimately supporting both intestinal and hepatic recovery (181). Together, these animal studies underscore the mechanistic plausibility of the gut barrier stabilizing effects observed in human data, and highlight the role of omega-3 PUFAs in modulating epithelial junction proteins, mucosal immune responses, and key inflammatory signaling networks.

### 5.6 Polyphenols

Polyphenols, a diverse group of plant-derived compounds abundant in fruits such as berries and grapes, vegetables like spinach and broccoli, and beverages like green tea, are potent antioxidants known for their anti-inflammatory properties. These compounds have garnered attention for their potential therapeutic effects in managing MASLD. Many reviews and meta-analyses have studied the effects of polyphenols on MASLD, showing promising results but also some inconsistencies that require further research.

A comprehensive meta-analysis assessed the impact of eight different polyphenolic compounds, such as curcumin, resveratrol, naringenin, anthocyanins, hesperidin, catechins, silymarin, and genistein, in patients with MASLD. Curcumin was found to significantly reduce BMI, liver enzymes (AST, ALT), TG, TC, and HOMA-IR without adverse effects. Similarly, naringenin reduced TG, TC, and LDL-C levels and enhanced HDL-C, though it had minimal impact on liver enzyme activity. Catechin and hesperidin also improved metabolic parameters, while silymarin effectively reduced hepatic fat accumulation and liver stiffness (182). However, the overall analysis indicated inconsistencies among randomized controlled trials (RCTs), necessitating further studies.

Another systematic review examined 29 RCTs involving 1,840 patients and confirmed that curcumin and turmeric supplementation improved liver enzymes, inflammatory cytokines, lipid profile, and insulin resistance. Silymarin consistently reduced liver enzymes and lipids but did not significantly alter inflammatory markers. Resveratrol showed inconsistent results, while hesperidin and naringenin improved lipid and liver enzyme profiles (183). These findings suggest that polyphenol supplementation may aid liver disease management, but optimal dosages and treatment durations remain undetermined.

A cross-sectional study assessed the correlation between polyphenol intake and disease prevalence in a large cohort of 9,894 adults. Increased consumption of total polyphenols, phenolic acids, and lignins was linked to a reduced risk of liver disease, especially in women. However, total flavonoid intake did not show a significant association (184). These results suggest that polyphenol-rich diets could play a preventive role, but interventional studies are needed to confirm causality.

Polyphenols are essential in influencing the balance of intestinal microbiota and supporting the integrity of the gut barrier, which is essential for overall health, including the management of MASLD and MASH. Certain polyphenolic compounds, including chlorogenic acid, curcumin, catechins from green tea, naringenin, quercetin, and resveratrol, have been found to beneficially affect gut health and the gut-liver interaction, providing potential therapeutic avenues for managing metabolic disorders.

Chlorogenic acid (CGA), a compound found in coffee, supports metabolic health by influencing gut microbiota and liver function. In preclinical MASH models, CGA improves liver health by reducing liver and blood lipids and inflammation. This effect is largely attributed to CGA’s ability to regulate the gut microbiota, promoting the growth of beneficial bacteria like *Bacteroides* and *Akkermansia*, while inhibiting harmful species like *Firmicutes* (185). In a separate study on high fat diet induced MASLD in mice, CGA administration not only alleviated hepatic steatosis and inflammation but also reduced serum transaminase levels, fasting blood glucose, and blood lipids. Additionally, CGA reversed the activation of the TLR4 signaling pathway and reduced inflammatory cytokines, including TNF-α, IL-1, IL-6, and TNF-β, in the liver. This effect was linked to an increase in gut microbiota diversity, particularly promoting *Bifidobacterium* and reducing *Escherichia coli*. Furthermore, CGA enhanced intestinal barrier integrity by upregulating tight junction proteins, such as Occludin and ZO-1, in intestinal tissue (186). Lastly, in a high L-carnitine feeding mouse model, CGA was found to inhibit the formation of trimethylamine-N-oxide (TMAO), a compound known to contribute to liver dysfunction. By reducing TMAO levels, CGA mitigated liver injury markers and inflammation. The gut microbiota composition was also remodeled, with an increase in beneficial bacteria like *Bacteroides* and *Akkermansia*, and a decrease in harmful species such as *Firmicutes* and *Erysipelotrichaceae*. This microbiota remodeling, combined with CGA’s antioxidant and anti-inflammatory properties, contributed to improved liver health and reduced liver enzyme levels (AST, ALT) (187).

Curcumin, a polyphenolic compound derived from *Curcuma longa*, is known for its antioxidant, anti-inflammatory, and hepatoprotective properties. Studies have demonstrated that curcumin modulates the gut microbiota by increasing beneficial bacteria such as *Lactobacillus* and reducing harmful species like *Proteobacteria* (188, 189). In a high-fat diet (HFD)-induced rat model of MASLD, curcumin alleviated hepatic steatosis and metabolic endotoxemia, and improved intestinal barrier integrity by upregulating tight junction proteins such as Occludin and ZO-1 (188). In a comparative study between curcumin and metformin in HFD-induced obesity in rats, both compounds were found to attenuate hepatic fat accumulation, reduce the *Firmicutes/Bacteroidetes* ratio, increase the abundance of beneficial bacteria including *Butyricicoccus* and *Lactobacillus*, and decrease opportunistic pathogens. These microbiota changes were accompanied by improvements in metabolic parameters and reductions in inflammatory cytokines, such as IL-6, IL-1β, and TNF-α (189).

Green tea catechins, particularly epigallocatechin gallate (EGCG), are well-known for their antioxidant properties and potential to modulate gut microbiota. In mouse models of MASH, EGCG supplementation has been shown to significantly alter the intestinal microbiota composition. Specifically, EGCG promotes the growth of beneficial bacteria such as *Akkermansia* while reducing harmful species, including *Firmicutes* (190, 191). Additionally, EGCG has been found to improve liver health by reducing liver triglyceride content and fatty lesions, as well as alleviating dysregulated bile acid metabolism. This effect is associated with a shift in microbial composition, with EGCG increasing the abundance of genera like *Adlercreutzia* and *Allobaculum*, and decreasing the abundance of *Desulfovibrionaceae* (192). Moreover, EGCG enhances gut barrier function by influencing gut microbiota and bile acid metabolism, particularly by improving the balance of primary and conjugated bile acids. It has been suggested that EGCG’s beneficial effects on liver health are mediated, in part, through microbiota-driven changes in bile acid composition, which can contribute to the suppression of hepatic steatosis (191, 192).

Naringenin, a flavonoid found in citrus fruits, positively influences gut microbiota by promoting the growth of beneficial bacteria such as Allobaculum and reducing harmful species like *Fusobacterium* (193). It also enhances gut barrier integrity, reduces liver fat accumulation, and alleviates inflammation. In a study conducted on C57BL/6J mice, naringenin supplementation reduced body weight gain, liver fat accumulation, and lipogenesis, while improving plasma biochemical parameters in high-fat diet-fed mice. Analysis of the gut microbiota showed that naringenin altered the microbial community composition, increasing beneficial bacteria and decreasing harmful ones. Spearman’s correlation analysis revealed that bacteria such as *Allobaculum*, *Alloprevotella*, and *Butyricicoccus* were negatively correlated with serum lipid levels, while *Campylobacter, Faecalibaculum*, and *Fusobacterium* showed positive correlations (193). Further studies indicate that naringenin enhances intestinal barrier function by promoting the expression and cytoskeletal association of tight junction proteins in Caco-2 cells. Naringenin significantly increased the expression of claudin-4 and other tight junction proteins such as occludin and claudin-1, which are essential for maintaining intestinal integrity (194). Additionally, naringenin supplementation in colitic mice demonstrated protective effects on the intestinal barrier, attenuating the effects of dextran sulfate sodium (DSS) induced colitis by reducing inflammation and improving tight junction integrity. Naringenin supplementation attenuated the increase in colonic permeability and prevented the disruption of tight junction protein expression, including occludin and claudin-3, which are critical for maintaining the intestinal epithelial barrier. By stabilizing these TJ proteins, naringenin reinforced the intestinal barrier function. Additionally, naringenin reduced the expression of inflammatory cytokines, such as IL-6 and IL-17A, and mitigated the clinical and histopathological signs of colitis, suggesting its dual role in both barrier protection and inflammation modulation (195).

Quercetin, a flavonoid found in fruits and vegetables, has demonstrated beneficial effects on gut microbiota composition and liver health, particularly in animal models of MASLD. In mice fed a high-fat diet, quercetin and its glycoside derivatives—especially isoquercetin—promoted the growth of beneficial bacteria such as *Lactobacillus*, *Bifidobacterium*, and *Akkermansia*, while counteracting dysbiosis commonly associated with disease progression (196). These microbial changes were accompanied by improved gut barrier function and reduced levels of systemic inflammation. In the same murine model, quercetin administration led to a reduction in plasma LPS levels and suppression of TLR4–NF-κB signaling, indicating decreased gut-derived endotoxemia and leaky gut-associated inflammation (197). In a separate rat model of early-stage MASLD, quercetin treatment was associated with reduced body fat accumulation, improved hepatic lipid profile, modulation of inflammatory gene expression, and restoration of gut microbiota composition (198). Several studies have highlighted the beneficial effects of quercetin on intestinal epithelial integrity. *In vitro* experiments using human intestinal epithelial Caco-2 cells demonstrated that quercetin enhances barrier function by increasing transepithelial resistance and promoting the expression and membrane localization of tight junction proteins such as claudin-4, occludin, and ZO-2 (199, 200). These effects were associated with a reduction in paracellular permeability and involved modulation of the PKCδ signaling pathway (200). In aged breeder chickens, the combination of dietary quercetin and vitamin E improved intestinal morphology and reduced serum markers of barrier disruption, such as D-lactate and diamine oxidase, while enhancing the expression of mucosal tight junction proteins including occludin, claudin-1, and ZO-1 (201). Furthermore, in a mouse model of fructose-induced hepatic steatosis, quercetin supplementation not only reduced hepatic lipid accumulation and inflammatory cytokine levels but also led to favorable shifts in gut microbiota composition such as a decreased *Firmicutes/Bacteroidetes* ratio and increased abundance of Parabacteroides and Alloprevotella. These changes were accompanied by elevated levels of propionic acid, suggesting enhanced microbial fermentation activity and improved gut barrier function (202).

Resveratrol, found in grapes and berries, exhibits anti-inflammatory and antioxidant properties. Recent studies suggest that its beneficial effects on gut microbiota and intestinal barrier integrity may contribute to the amelioration of hepatic steatosis and metabolic disturbances. In a study using a mouse model of MASLD, resveratrol supplementation led to beneficial alterations in the gut microbiota. Specifically, treatment increased the abundance of *Bacteroidetes* and *Bifidobacterium*, while reducing *Firmicutes*. Functional metagenomic analysis based on 16S rRNA sequencing revealed a downregulation of microbial genes involved in lipid and glucose metabolism, suggesting a shift toward a metabolically favorable gut microbial profile (203). A comparative study employing animal models investigated the effects of resveratrol and its glycosylated precursor, polydatin, on gut microbiota-derived metabolites. The study reported increased levels of short-chain fatty acids (SCFAs), particularly valeric and caproic acid, in the feces of treated animals. These SCFAs were associated with activation of AMP-activated protein kinase (AMPK), an important regulator of lipid metabolism, indicating that resveratrol and its derivatives may improve hepatic lipid handling via microbiota-mediated SCFA production (204). In another mouse study modeling high fat diet induced MASLD, resveratrol administration improved intestinal barrier function. This was evidenced by enhanced intestinal morphology and increased expression of tight junction proteins such as ZO-1 and occludin. These changes were accompanied by reduced serum levels of LPS, a biomarker of increased intestinal permeability, and attenuated liver inflammation, suggesting that resveratrol reduces endotoxemia and systemic inflammation by restoring gut barrier integrity (205). A recent animal study investigated how resveratrol improves MASLD in high fat diet fed mice. Treatment reduced body weight, hepatic steatosis, and insulin resistance, while enhancing gut barrier integrity by improving mucosal morphology and increasing barrier protein expression. The study also showed resveratrol modulated gut microbiota, suppressing harmful bacteria (e.g., *Desulfovibrio*, *Alistipes*) and enriching SCFA-producing bacteria (e.g., *Allobaculum*, *Bacteroides*). Microbiota transfer from resveratrol-treated mice to untreated mice reproduced these benefits, highlighting a microbiota-mediated mechanism. However, low-dose resveratrol was less effective in preventing steatohepatitis, indicating dose dependency in its therapeutic effects (206).

### 5.7 Probiotics

Probiotics are live bacteria that provide health benefits if taken in sufficient quantities. These helpful microorganisms are typically present in fermented products such as yogurt, kefir, kimchi, miso, and some cheeses. Additionally, probiotics are available in dietary supplements and pharmaceuticals that promote gut health (207). The gut microbiota, consisting of trillions of microorganisms, plays a key role in maintaining metabolic and immune balance. Its composition is influenced by various factors, including diet, environment, and early microbial exposure during birth (208, 209). Major bacterial phyla include *Firmicutes* and *Bacteroidetes*, with archaea primarily represented by *Euryarchaeota* (210).

Probiotics help modulate this microbiota composition, strengthen the intestinal barrier, and regulate immune functions (211). Several animal studies have demonstrated the beneficial effects of probiotics in the context of MASLD. In a murine model of MASLD/MASH induced by high-fat diet, oral administration of a proprietary probiotic mixture, Prohep, was shown to alleviate hepatic steatosis, improve plasma lipid profiles, and reduce hepatic lipogenesis and cholesterol biosynthesis gene expression. Prohep also modulated the gut microbiota, altered bile acid profiles, and increased fecal SCFAs. In a prolonged high fat diet model, Prohep further ameliorated hepatic inflammation and fibrosis, indicating potential in preventing MASLD progression to MASH (212). In a separate rat model, probiotic rich yogurt containing *Lactococcus rhamnosus*, *Lactobacillus plantarum*, *Lactobacillus acidophilus*, and *Bifidobacterium lactis* significantly attenuated high fat diet induced MASLD. Animals treated with the multi-strain yogurt exhibited improved metabolic and liver function markers, including reduced cholesterol, triglycerides, LDL, glucose, insulin, and ALT levels. Liver histology showed near-normal architecture with reduced steatosis. Notably, the probiotic formulation also improved the gut-liver axis by lowering coliform and *Staphylococcus* counts in the small intestine (213). *In vitro* mechanistic studies further support these findings. A human “leaky gut-on-a-chip” model mimicking cytokine-induced epithelial barrier dysfunction demonstrated that probiotic administration restored tight junction protein expression (ZO-1, occludin), enhanced mucus production, and reduced pro-inflammatory signaling pathways (p65, pSTAT3, MYD88). This model offers insight into the barrier restorative effects of probiotics under inflammatory conditions. Similarly, in LPS challenged piglets, *Lactobacillus* supplementation improved intestinal histology, upregulated tight junction proteins, reduced pro-inflammatory cytokines (IL-1β, IL-6, TNF-α), and increased anti-inflammatory mediators (IL-10, Arg1). These effects were partially mediated through the modulation of macrophage phenotypes and enhanced SCFA signaling via GPR43 (214).

Despite these promising results from animal and *in vitro* studies, the clinical evidence for the efficacy of probiotics in treating MASLD remains inconsistent. Several randomized, double-blind, placebo-controlled studies have assessed the effects of probiotics on liver function, metabolic parameters, and disease severity. In one set of studies, probiotics were found to reduce the percentage of patients with moderate and severe fatty liver, but they did not significantly improve liver enzyme levels such as ALT, AST, TG, or insulin IR (215, 216). This suggests that while probiotics might help in modulating the progression of fatty liver, their effects on key metabolic markers remain inconsistent. Nevertheless, other clinical trials have reported more favorable outcomes. However, other clinical trials have provided more positive results. A 3-month study demonstrated that probiotic supplementation significantly improved several liver function markers. Additionally, the probiotic group showed better fecal flora conditions compared to the placebo group, indicating potential benefits for liver function, glucose, and lipid metabolism in MASLD patients (217). Similarly, a 12-week study showed significant reductions in TC, ALT, AST, GGT, and alkaline phosphatase (ALP) levels following probiotic intervention (218).

Meta-analysis studies also provide valuable insights. A meta-analysis involving 21 randomized clinical trials with 1,037 MASLD patients, demonstrated that probiotics significantly reduced liver enzyme levels, blood lipid concentrations, and markers of glucose metabolism. This suggests a beneficial role of probiotics in improving hepatic steatosis. However, the study noted that probiotics did not significantly affect BMI, inflammatory markers, or insulin resistance, highlighting the need for more research into the long-term metabolic effects of probiotics (219). Another systematic review involving 34 studies with 12,682 individuals identified beneficial effects of probiotics on liver health, lipid regulation, and inflammatory markers in individuals with MASLD. The study reported significant reductions in markers of hepatic fibrosis, ALT, AST, ALP, TG, and TNF-α (220). These findings suggest that microbiota-targeted therapies, including probiotics, could contribute to delaying the advancement of MASLD.

## 6 Disruption of intestinal barrier integrity and its role in the progression of MASLD

Maintaining the integrity of the gut epithelial barrier is essential in regulating immune responses and modulating inflammation. Epithelial barrier dysfunction, microbial translocation, immune cell activation, and disruption of tight junction proteins are among the processes involved in MASLD (**Table 2**). In healthy states, this barrier prevents the movement of microbial substances from the gut into the bloodstream, which helps control immune system activation. In MASLD, a weakened barrier allows microbial molecules like LPS to pass through and reach the portal circulation. Once in the liver, these microbial components serve as potent inducers of hepatic inflammation. In MASLD, disruption of this epithelial architecture facilitates microbial translocation and promotes systemic and hepatic inflammation (221, 222).

**Table 2:** Mechanisms linking disruption of intestinal barrier in MASLD.

Process/Mechanism	Description	Key Findings/References
Intestinal Epithelial Barrier Dysfunction	Impaired integrity of tight junctions (ZO-1, occludin) increases permeability, allowing microbial components like LPS to enter the bloodstream.	- Reduced ZO-1 and occludin expression in MASLD patients (223) - Increased circulating LPS in steatosis and MASH patients (224)
Gut Microbiota Dysbiosis	Alterations in gut microbiota composition increase the abundance of gram-negative bacteria, leading to enhanced LPS production.	- Diets rich in saturated fats and fructose induce microbial dysbiosis and increased LPS production (110, 227, 228)
Immune Activation	Increased microbial components activate immune responses, including proinflammatory cytokine release and immune cell imbalance.	- Reduced regulatory T cells (FoxP3+ Tregs) and increased Th1/CD8+ T cells (13, 235) -Intestinal B cell activation (236, 237)
Histamine and Mast Cells	Mast cells release histamine, cytokines, and proteases (e.g., tryptase, chymase), increasing intestinal permeability and promoting bacterial translocation.	- Histamine increases gut permeability and induces translocation (238, 239)
Liver Inflammation and Fibrosis	LPS and microbial metabolites activate Kupffer cells and hepatic stellate cells, promoting inflammation and fibrosis.	- TLR-4 activation and upregulation of fibrogenic mediators (TGF-β1, α-SMA) (230, 231)
Gut-Vascular Barrier (GVB) Impairment	Altered GVB increases intestinal permeability, allowing LPS to reach the liver, further promoting inflammation.	- Increased expression of PV-1 in MASLD patients (232) - Disruption of WNT/β-catenin signaling enhances LPS dissemination (233, 235)

Clinical studies have consistently demonstrated impaired intestinal barrier integrity in MASLD. Reduced expression of TJ proteins (ZO-1 and occludin) in intestinal epithelial cells has been documented in MASLD patients, particularly in those with elevated serum transaminases, and inversely correlates with ALT and AST levels, suggesting that epithelial barrier dysfunction is associated with hepatocellular injury (223). A recent meta-analysis further confirmed significantly elevated circulating LPS levels in both simple steatosis and MASH patients, which correlated with histological severity, serum C-reactive protein (CRP), and markers of insulin resistance (224). These findings suggest that circulating LPS may serve as both a surrogate biomarker of increased intestinal permeability and an active driver of systemic and hepatic inflammation in MASLD. In addition, human studies using intestinal permeability tests (e.g., 51Cr-EDTA or lactulose-mannitol ratio) and duodenal TJ protein expression analyses demonstrated significantly increased gut permeability and reduced ZO-1 or claudin expression in MASLD patients compared to healthy controls, even at early disease stages. These findings underscore the critical role of TJ dysfunction in MASLD progression, with alterations in TJ integrity directly correlating with disease severity (225, 226).

Experimental models of MASLD provide mechanistic support for the causal role of gut barrier dysfunction in disease pathogenesis. Diets rich in saturated fats and fructose induce intestinal dysbiosis characterized by an increased abundance of gram-negative bacteria and a reduction in beneficial commensals (e.g., *Firmicutes* and *Akkermansia muciniphila*), leading to enhanced LPS production and compromised epithelial renewal (110, 227, 228). Concomitant reductions in goblet cell density and mucin secretion further impair the mucosal defense layer, facilitating microbial interaction with epithelial surfaces (227). Proteomic analysis of small intestinal epithelial cells from high-fat diet-fed mice revealed downregulation of TJ-associated proteins, including claudin-7 (Cldn7), and disruption of the EpCAM/Cldn7 complex, linking diet-induced molecular changes directly to increased gut permeability (229).

LPS translocation activates hepatic TLR-4 signaling, promoting the expression of proinflammatory cytokines (e.g., TNF-α, IL-6) and fibrosis-related mediators (e.g., TGF-β1, α-SMA) (230, 231). TLR-4 activation has also been shown to upregulate hepatic glutaminase 1 (GLS), increasing intrahepatic ammonia production, which exacerbates mitochondrial dysfunction, oxidative stress, and hepatocellular injury. Recent studies demonstrated that pharmacological inhibition of TLR4 or GLS attenuates hepatic inflammation and fibrosis and reduces systemic ammonia levels in both *in vitro* hepatocyte cultures and murine models of MASLD (62).

Beyond epithelial integrity, the gut-vascular barrier (GVB) a specialized endothelial interface regulating the passage of macromolecules into the portal circulation is also impaired in MASLD. Increased expression of plasmalemma vesicle-associated protein-1 (PV-1), an indicator of endothelial hyperpermeability, has been detected in intestinal tissues of MASLD patients (232). Experimental disruption of WNT/β-catenin signaling, a pathway essential for GVB maintenance, results in LPS dissemination into the portal vein and exacerbates hepatic inflammation in murine models fed Western diets (233, 234). These findings suggest that combined epithelial and vascular barrier dysfunction underlies the endotoxemic phenotype observed in MASLD.

The disruption of epithelial barrier integrity is associated with both microbial translocation and immune cell imbalances. This condition is particularly characterized by a reduction in regulatory T cells (FoxP3^+^ Tregs) and an increase in Th1 and CD8^+^ T cells within the lamina propria (13, 235). These immunological alterations contribute to the formation of a pro-inflammatory microenvironment, which further compromises epithelial integrity and facilitates microbial translocation. In recent years, findings have suggested that B cells originating from the gut microbiota may also play a role in the development of liver inflammation and fibrosis. Single-cell RNA sequencing analyses performed in MASH model mice have revealed that intrahepatic B cells exhibit a pro-inflammatory transcriptional profile. This suggests that these cells are activated in a manner that supports hepatic inflammation. Consistent with these findings, B cell depletion has been reported to reduce MASH progression, whereas the adoptive transfer of B cells isolated from MASH livers was shown to recapitulate disease features in recipient mice. Additionally, fecal microbiota transplantation from MASLD patients into recipient mice has been shown to promote intrahepatic B cell accumulation and activation, thereby facilitating MASH progression (236). In another study, an increase in active B cells was observed in the gastrointestinal tissues of both MASH model mice and MASH patients, while B cell depletion was shown to prevent or reverse MASH pathology and liver fibrosis. It has been proposed that IgA secretion activates CD11b^+^CCR2^+^F4/80^+^CD11c^−^FcγR1^+^ hepatic myeloid cells, thereby initiating fibrotic processes. Indeed, in MASH patients, a positive correlation has been reported between IgA levels, hepatic myeloid cell activation, and the degree of liver fibrosis (237). Mast cells residing in the intestinal mucosa also play a critical role in this process by releasing histamine, cytokines, and proteases such as tryptase and chymase, thereby promoting increased intestinal permeability. These proteases have been reported to degrade TJ proteins and suppress the expression of junctional adhesion molecule A, leading to enhanced epithelial permeability (238). Experimental models have demonstrated that histamine directly increases gut permeability and induces bacterial translocation, thereby promoting systemic inflammation (239).

**Figure 3:**
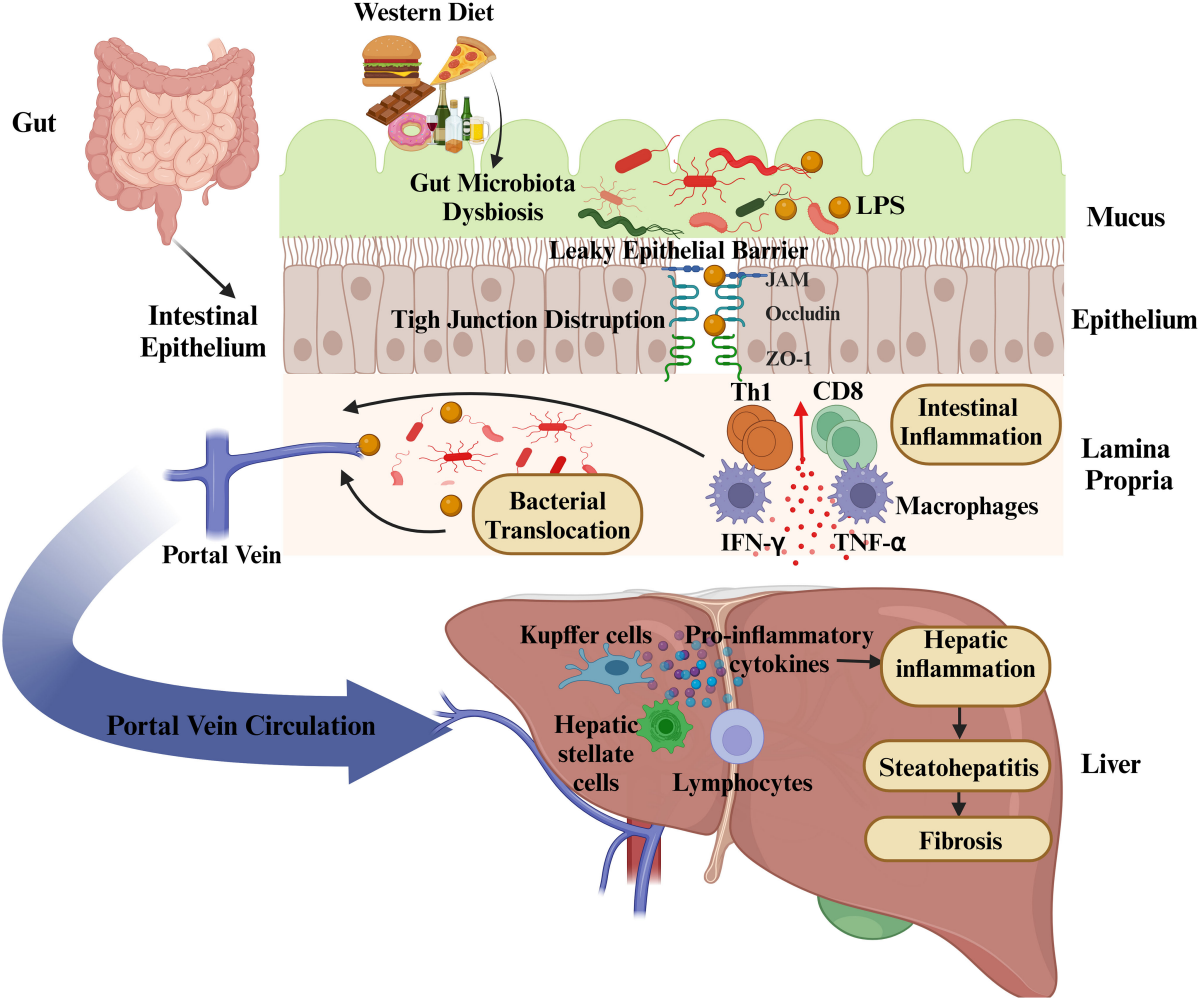
Disruption of the intestinal epithelial barrier and its impact on metabolic dysfunction-associated steatotic liver disease (MASLD) progression. A Western diet alters gut microbiota composition, leading to dysbiosis and increased intestinal permeability due to tight junction disruption (occludin, ZO-1, JAM). This facilitates bacterial translocation and the release of lipopolysaccharides (LPS) into the portal circulation, triggering immune activation. In the gut, Th1 and CD8^+^ T cells promote inflammation through interferon-gamma (IFN-γ) and tumor necrosis factor-alpha (TNF-α) secretion. In the liver, Kupffer cells and hepatic stellate cells respond to microbial products by releasing proinflammatory cytokines, recruiting lymphocytes, and exacerbating hepatic inflammation. These processes contribute to the progression of MASLD, promoting steatohepatitis and fibrosis.

The disruption of the intestinal epithelial barrier initiates a cascade of events that contribute to the progression of MASLD. The weakening of tight junction proteins (occludin, ZO-1, JAM) increases intestinal permeability, allowing microbial products particularly LPS to enter the systemic circulation. Alterations in the gut microbiota amplify this cycle, leading to increased local and systemic immune activation, which accelerates disease progression. In the gut, Th1 and CD8+ T cells produce proinflammatory cytokines that contribute to the inflammatory response. LPS, as a pathogen-associated molecular pattern, is recognized by the immune system, activating Kupffer cells and hepatic stellate cells in the liver. This activation enhances inflammation and drives the transition from steatosis to steatohepatitis and fibrosis (**Figure 3**). The gut-liver axis plays a crucial role in MASLD progression, suggesting that targeting the intestinal barrier and restoring microbial balance may offer potential therapeutic strategies to halt disease progression.

## 7 Therapeutic strategies targeting the gut-liver axis

The management of MASLD remains challenging due to the limited options for targeted treatments. While numerous strategies focus on lifestyle modifications and pharmacotherapy, further research is needed to establish effective treatments. Recent advancements in MASLD research highlight the gut-liver axis as a key therapeutic target (**Table 3**).

**Table 3:** Therapeutic strategies targeting the gut-liver axis in MASLD.

Therapeutic Strategy	Mechanism	Impact on MASLD	References
Dietary Interventions	Balanced diet focusing on whole foods, healthy fats, and lean proteins, reducing refined carbohydrates, unhealthy fats, and cholesterol. Promotes beneficial shifts in the gut microbiota.	Inflammation reduction and insulin sensitivity improvement. Gut barrier integrity enhancement and microbial leakage reduction.	(66, 240–244)
Physical Exercise	Aerobic and resistance training improve liver function and enhance gut barrier integrity. Regular physical activity promotes beneficial gut microorganisms.	Hepatic steatosis reduction and systemic inflammation alleviation, leading to liver health improvement. Potential risks associated with excessive training.	(245–251)
Gut Microbiome Modulation	Probiotics and other microbiome-modulating therapies aim to restore balance in gut bacteria and improve metabolic markers.	Decreased liver fat accumulation and alleviation of systemic inflammation, supporting gut barrier function. Reduction in liver cholesterol and total cholesterol (TC) levels.	(252–255)
Fecal Microbiota Transplantation (FMT)	FMT from healthy donors can restore gut health, potentially improving liver function.	Liver steatosis and inflammation reduction in MASLD animal models. Clinical outcomes remain inconclusive.	(256–260)
Bacteriophage Therapy and Synthetic Live Bacterial Therapeutics	Phage therapy targets pathogenic bacteria while preserving beneficial gut microbiota. Engineered probiotics like SYNB1020 metabolize toxic compounds.	Enhancement of metabolic and inflammatory markers in preclinical models.	(261, 262)

### 7.1 Dietary interventions

Dietary habits are fundamental in both the development and management of chronic liver disease. Studies suggest that diets high in refined carbohydrates, unhealthy fats, and cholesterol contribute to the progression of MASLD. Conversely, diets abundant in whole foods, healthy fats, and lean proteins are associated with a lower risk of developing the condition (240, 241). One proposed regimen focuses on a balanced intake of carbohydrates from cereals, fruits, and vegetables, minimizing fat intake, and using vegetable-based fats. Importantly, alcohol consumption and smoking should be avoided to improve liver health.

Diet can also influence the gut microbiota, a critical factor in MASLD progression. A healthy diet promotes beneficial shifts in the microbiome, such as an increase in species like *Akkermansia* and *Bifidobacterium*, which are associated with improved gut barrier integrity, reduced microbial leakage into the bloodstream, and improved insulin sensitivity (242, 243). These beneficial shifts can enhance gut homeostasis and reduce systemic inflammation, which is pivotal in the treatment of MASLD (244). In a recent study, it has been highlighted that sulforaphane (SFN), a bioactive compound found in cruciferous vegetables like broccoli, reduces weight gain, hepatic inflammation, and steatosis in mice fed high-fat and fructose diets. The study reports that SFN alters the gut microbiota composition, increases the expression of the intestinal barrier protein ZO-1, reduces serum LPS levels, and suppresses the LPS/TLR4 and endoplasmic reticulum stress pathways. Consequently, intestinal inflammation is reduced, gut integrity is preserved, and microbial product translocation to the liver is prevented. Additionally, SFN was found to decrease LPS levels in the liver and the associated inflammatory response (66).

### 7.2 Physical exercise

Physical activity, particularly aerobic and resistance training, has shown significant benefits in reducing hepatic steatosis and alleviating cardiovascular risks associated with MASLD (245, 246). Exercise not only improves liver function directly but also has systemic effects, such as enhancing gut barrier integrity. Studies suggest that regular physical activity promotes the proliferation of beneficial gut microorganisms, enhances immune system function, and reduces oxidative stress (247, 248). Animal studies have indicated that exercise can restore gut-liver axis balance by preserving the intestinal barrier, thereby preventing inflammation and improving liver health (249, 250). Nevertheless, recent experimental data indicate that excessive training may exert deleterious effects on the liver. In a murine model, overtraining-induced accumulation of lactate in skeletal muscle promoted the lactylation and phase separation of SORBS3, leading to the formation of lactate-enriched extracellular vesicles (“lactate bodies”) that triggered hepatocyte apoptosis and hepatic stellate cell activation via the MCL1–BAX/BAK signaling pathway (251).

### 7.3 Gut microbiome modulation

Restoring the balance of the gut microbiota is another therapeutic avenue for MASLD. Dysbiosis, or microbial imbalance, is a hallmark of liver diseases, and recent studies have suggested that correcting this imbalance could alleviate MASLD symptoms. Clinical and preclinical studies have explored the use of probiotics, demonstrating their potential in reducing liver fat accumulation and improving metabolic markers (252, 253). For instance, combining *Bifidobacterium* and *Lactobacillus* strains has demonstrated a reduction in liver cholesterol and TC levels. Moreover, probiotics may help restore intestinal barrier function and reduce systemic inflammation, further supporting their potential in MASLD management (254). Probiotics influence intestinal barrier function by regulating the expression of genes and proteins involved in TJ signaling in intestinal epithelial cells. *In vitro* and *in vivo* studies have reported that probiotics enhance the expression of ZO1, occludin, and claudin1, thereby strengthening the intestinal barrier and providing protection against infections (255).

### 7.4 Fecal microbiota transplantation

Fecal microbiota transplantation (FMT) offers a novel method for improving gut health, potentially leading to better liver function. Animal studies have demonstrated that FMT from healthy donors can reduce liver steatosis and inflammation in MASLD models (256, 257).

However, clinical trials investigating FMT for MASLD and related metabolic conditions have yielded mixed results. While some studies report improvements in gut permeability and reductions in liver fat accumulation, the clinical outcomes are still inconclusive (258–260). A randomized, double-blind, placebo-controlled trial conducted in adolescents with obesity (n = 87) evaluated the effects of a single-dose oral FMT from lean donors. Although no significant effect was observed on BMI standard deviation scores at six weeks, a reduction in android-to-gynoid fat ratio was reported at weeks 6, 12, and 26. Moreover, *post hoc* analyses suggested greater resolution of undiagnosed metabolic syndrome in the FMT group compared to placebo (258). In a pilot study involving adults with obesity and mild-to-moderate insulin resistance, participants received weekly oral FMT or placebo capsules for six weeks. Despite successful engraftment of donor microbiota persisting up to 12 weeks, no significant improvements were observed in insulin sensitivity (measured via hyperinsulinemic-euglycemic clamps), body composition, or glycemic markers (259). A separate randomized clinical trial directly targeting MASLD patients compared heterologous FMT (administered via colonoscopy and enemas) to probiotic therapy, with both groups adhering to lifestyle modifications. FMT was associated with a reduction in hepatic fat accumulation and improved gut microbiota composition. Notably, the therapeutic effect was more pronounced in lean MASLD patients compared to their obese counterparts (260). While these findings highlight the potential of FMT to modulate gut microbiota and influence metabolic parameters, the clinical efficacy in MASLD remains inconclusive. Variations in study design, route of administration, patient phenotypes, and follow-up durations may account for the inconsistent outcomes. Further large-scale, standardized clinical trials are warranted to evaluate the long-term impact of FMT in MASLD patients.

### 7.5 Bacteriophage therapy and synthetic live bacterial therapeutics

Bacteriophage therapy offers a targeted approach to eliminating pathogenic gut bacteria that contribute to MASLD progression. Specific phage cocktails are being developed to selectively modulate gut microbiota without disrupting beneficial bacterial populations (261). Similarly, engineered probiotics, such as SYNB1020, have been designed to metabolize toxic intestinal compounds, improving metabolic and inflammatory markers in preclinical models (262).

## 8 Conclusion remarks

The progression of MASLD is intricately shaped by a web of factors that extend beyond the liver itself. Central to its pathogenesis is the breakdown of the intestinal epithelial barrier, a key player in maintaining gut homeostasis. When this barrier is compromised, intestinal permeability increases, allowing harmful microbes and toxins to infiltrate the bloodstream. This not only exacerbates liver inflammation and fibrosis but also affects systemic health, linking gut dysfunction to extrahepatic manifestations of the disease. Dysregulation in tight junctions, microbiome imbalances, and immune activation at the gut-liver axis collectively drive the progression of MASLD.

The gut microbiota is central to this complex system, serving as both a mediator and regulator of immune responses. A well-balanced gut flora is crucial for establishing immune tolerance and supporting defense mechanisms, thereby maintaining the health of both the gut and liver. However, dysbiosis disrupts this balance, leading to heightened inflammation, metabolic dysfunction, and further epithelial barrier damage. The intimate relationship between the immune system, gut microbiota, and epithelial integrity underscores the need for a comprehensive approach to MASLD that includes both immune modulation and gut health restoration.

While therapeutic strategies, including dietary modifications and physical exercise, have shown promising results in improving liver function and microbiome composition, much remains to be understood. The potential of FMT as a therapeutic approach is still unclear, with varying clinical results emphasizing the necessity for more research on the mechanisms of microbiota-host interactions. The future of MASLD management may lie in therapies that restore the balance between the immune system, the gut microbiota, and epithelial integrity, unlocking new pathways to alleviate both hepatic and extrahepatic manifestations of this increasingly prevalent disease.
